# Development and application of KASP assays to differentiate between *Sorghum bicolor* , *halepense* , and their hybrids

**DOI:** 10.1002/ps.70618

**Published:** 2026-02-06

**Authors:** Connor Purvis, Eric L. Patterson, Erin E. Burns

**Affiliations:** ^1^ Department of Plant, Soil and Microbial Sciences Michigan State University East Lansing MI USA

**Keywords:** crop‐to‐weed introgression, hybrid detection, KASP assay, polyploidy, *Sorghum halepense*

## Abstract

**BACKGROUND:**

*Sorghum bicolor* and *Sorghum halepense* can readily hybridize, creating difficulty in identification. No genetic tools exist to accurately distinguish *S. bicolor*, *S. halepense*, and their hybrids. Detecting hybridization is essential to monitor crop‐to‐weed introgression. This study utilizes a single nucleotide polymorphism (SNP) in the internal transcribed spacer region between *S. bicolor* and *S. halepense*. This SNP was utilized in Kompetitive allele‐specific PCR (KASP) assay to identify *S. bicolor*, *S. halepense*, and their hybrids.

**RESULTS:**

KASP assays were successful in accurately differentiating between *S. bicolor*, *S. halepense*, and their hybrids. The KASP assay performed as well as Oxford Nanopore sequencing for measuring SNP frequency and thus is a perfect proxy for genotyping. Greenhouse crosses confirmed crop‐to‐weed introgression, with *S. halepense* being more receptive to interspecific pollen. Known and unknown samples assayed displayed misidentification in germplasm lines and significant hybrid frequency in naturally occurring biotypes. Synteny analyses revealed duplications of the ITS region in *S. halepense*.

**CONCLUSION:**

We developed a novel KASP assay targeting a conserved SNP that accurately distinguishes between *S. bicolor*, *S. halepense*, and their hybrids. This assay was validated through Oxford Nanopore sequencing, greenhouse crosses, and diverse germplasm and natural collections. © 2026 The Author(s). *Pest Management Science* published by John Wiley & Sons Ltd on behalf of Society of Chemical Industry.

## Graphical abstract

A panel showing the morphological differences between *Sorghum halepense* (A), hybrids (B), and *Sorghum bicolor* (C) with a novel KASP‐based genetic assay capable of reliably distinguishing between them. Partially Created in BioRender. https://BioRender.com/1u30ukv

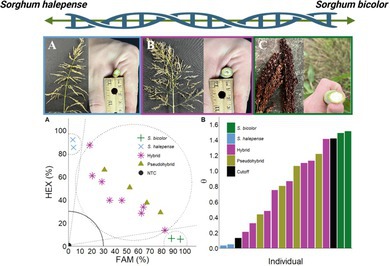

## 1 INTRODUCTION

Species hybridization has played a fundamental role in agronomic crop domestication by accelerating genetic improvements resulting in enhanced quality and higher yields.^1^, ^2^ While controlled hybrids have been essential for crop improvement, hybridization between crops and weeds is recognized as a driver of rapid evolution with potential negative consequences for control of formed hybrids.^3^ For instance, traits important for rice (*Oryza sativa* L. *ssp*.) and corn (*Zea mays* L. *ssp*. mexicana, *Zea luxurians*) cultivation have been shown to be transferred to weedy varieties and through introgression in several instances.^4^, ^5^ Further, there is evidence that these weed‐crop hybrids have increased fitness due to cultivated trait introgression.^6^ Therefore, the flow of agronomic traits into weeds has potential to increase weed resilience to stress.

The *Sorghum* genus is an important model for studying hybridization between crops and weeds. It consists of the domesticated crop grain sorghum (*Sorghum bicolor ssp. bicolor* (L.) Moench) and several destructive weeds including johnsongrass (*Sorghum halepense* (L.) Pers.) and shattercane (*Sorghum bicolor* (L.) Moench *ssp. verticilliflorum* (Steud.) de Wet ex Wiersema & J. Dahlb). *Sorghum bicolor* was domesticated approximately 6000 years ago^7^, ^8^ and is one of the most important cereal crops globally, with over 60 million metric tons produced annually.^9^ Species within this genus are reproductively compatible due to recent shared ancestry, overlapping phenology, and in some cases, compatible ploidy levels. Polyploidy plays a critical role in reproductive compatibility within *Sorghum*; *S. bicolor* is diploid (2n = 2x = 20), while *S. halepense* is a tetraploid (2n = 4x = 40). Despite these differences, repeated hybridization events have been shown between these species resulting in triploids, tetraploids, pentaploids, and hexaploids.^10^ Long‐term gene flow between the crop (*S. bicolor*) and its weedy relatives (*S. bicolor ssp. verticilliflorum* and *S. halepense*) has caused ambiguity in plant identification. Morphologically, hybrids are difficult to distinguish in the field due to taking on intermediate phenotypes between the parents.^11^ Further complicating the *Sorghum* genus is Columbus grass (*Sorghum almum* Parodi), a cultivated forage perennial hybrid, which can be mistaken for *S. halepense*.^12^

Climate change will exacerbate the current challenges arising from these hybridization events. Rising temperatures and potentially drier conditions will expand grain sorghum's range to more northern regions and increase its viability under water restrictions. It is well‐adjusted to climatic variability and low‐input environments, which will be useful as the climate continues to change.^13^ The range expansion for grain sorghum also includes expansion of its weedy relatives to these new ranges, which are also well adapted to climatic variability. Species distribution modeling indicates there will be a northward expansion of *S. halepense*, increasing opportunities for gene flow between the crop and weed.^14^ As ranges simultaneously expand, crop‐to‐weed introgression may increase in these closely related species.

As hybridization is known to happen between these species and their ranges will continue to overlap, a concerning aspect is the transfer of advantageous crop traits (increased growth rate, seed set, stress resistance, etc.) to weedy relatives. Additionally, the transfer of weedy traits such as herbicide resistance or abiotic stress tolerance to crops could make management difficult.^15^ Both *S. bicolor ssp. verticilliflorum* and *S. halepense* have known widespread cases of herbicide resistance to different sites of action.^16^, ^17^ Additionally, climate change has driven *S. bicolor* breeding to develop more drought‐tolerant lines which increases its potential to handle other abiotic stressors.^18^, ^19^ Pollen‐mediated gene flow has been shown at up to 50 m between *S. bicolor* and *S. halepense* biotypes, these traits associated with increased crop performance are at risk to increase weedy competitiveness.^20^

To effectively monitor gene flow dynamics and to better manage risks associated with crop‐weed hybridization, we need reliable tools to distinguish species and detect hybrids. There are currently no genetic markers that have been reported in the literature that can identify hybrids between *S. bicolor* and *S. halepense*. Kompetitive allele‐specific PCR (KASP) is a PCR‐based genotyping technique that uses single nucleotide polymorphisms (SNPs) for a wide range of applications. It has been used in marker‐assisted breeding in crops like rice (*Oryza sativa* L.) and wheat (*Triticum aestivum* L.) to identify valuable alleles^21^, ^22^ and has also been applied to identify interspecific hybrids in plants such as vanilla (*Vanilla spp*.) and watermilfoil (*Myriophyllum spp*.).^23^, ^24^ The KASP method is more cost‐effective than other genotyping methods. For example, Oxford Nanopore sequencing can cost approximately $15 per sample, whereas KASP assays can be run for one cent per sample using standard real‐time qPCR thermocyclers at scale.^25^

Therefore, the goal of this study was to develop a KASP‐based genetic assay capable of reliably distinguishing *S. bicolor*, *S. halepense*, and their hybrids. To achieve this goal, we had three objectives: (i) validate our marker and assay with known control samples from pure *S. bicolor* and *S. halepense* individuals and hybrids, (ii) obtain crosses between *S. bicolor*, *S. halepense*, and hybrid in the greenhouse to analyze F_1_ populations for hybrid formation, and (iii) assign genotypes to known germplasm biotypes and unknown naturally collected biotypes based on our KASP assay.

## 2 MATERIALS AND METHODS

### 2.1 Plant material for KASP

The Michigan State weed seed collection (Supporting Information, Table S1), hereby the MSU collection, was utilized to facilitate these experiments along with germplasm from the National Plant Germplasm System (NPGS) *Sorghum* collection (Supporting Information, Table S2). To investigate objective one, known *S. bicolor* and *S. halepense* biotypes along with a subset from the MSU collection that spanned a wide range of hybridization phenotypes were grown under greenhouse conditions in the spring of 2023 at MSU. Plants were grown under a 16‐h light period at 26 °C and an 8‐h dark period at 18 °C. At the three to four leaf stage, the newest leaf was collected, flash frozen in liquid nitrogen and stored at −80 °C until use. Seeds were only used for extraction if a biotype had poor germination. All tissue was collected in the same way for all experiments unless stated otherwise.

### 2.2 DNA extraction

DNA was extracted from *Sorghum* leaf or seed tissue (80 mg) using a modified CTAB protocol previously described.^26^ Tissue was first ground in liquid nitrogen with a mortar and pestle before being placed in a 2 mL centrifuge tube. In brief, 1% hot CTAB buffer (1 mL; 65 °C) with 2% ß‐mercaptoethanol was added to the tube and incubated for an hour at 65 °C. After incubation, samples were centrifuged for 2 min with the supernatant removed to a new tube and RNase (1 μL at 10 mg mL^−1^) added and incubated for 30 min at 37 °C. All centrifugation steps were performed at 15 000 rpm unless otherwise noted. Chloroform: isoamyl alcohol (600 μL; 24:1) was added and centrifuged for 3 min with the top layer removed to a new tube and chloroform: isoamyl alcohol (400 μL) added and centrifuged for three additional min. The top layer was removed (500 μL) to a new 2 mL centrifuge tube with 3 M sodium acetate (50 μL) and 100% EtOH (1 mL) added. Samples were precipitated by centrifugation for 1.5 min. A sequential pellet cleaning was performed with 70% EtOH (400 μL) for 3 min at room temperature. Excess ethanol was then removed with a Kim‐wipe, and the pellet was resuspended in nuclease‐free water (50 μL). DNA concentration and quality were quantified using a NanoDrop One spectrophotometer (Thermo Scientific, 5225 Verona Rd., Madison, WI 53711).

### 2.3 Primer design for KASP

**Figure 1:**
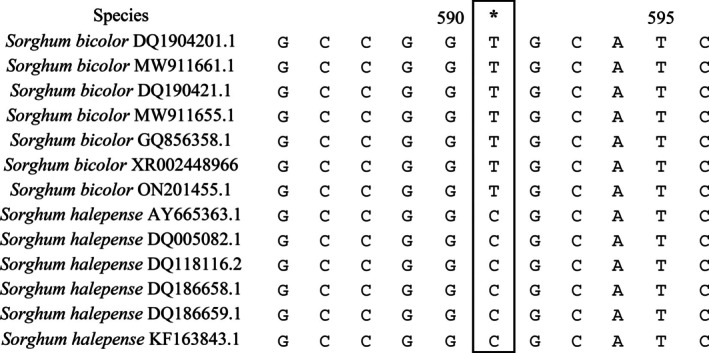
Distinguishing SNP at position 591 in the ITS region differentiating *Sorghum bicolor* (T) and *Sorghum halepense* (C). Asterisk and solid black square notes allele differences at position.

Thirteen sequences from the internal transcribed spacer (ITS) region, including seven from *S. bicolor* and six from *S. halepense*, were obtained from NCBI and aligned using MEGA 11^27^ to examine SNPs between the two species. A single SNP at position 591 (ITS‐SNP591) consistently differentiated the two species with *S. bicolor* accessions having a Thymine and *S. halepense* having a Cytosine at this position (Fig. 1). This SNP was utilized for designing allele‐specific KASP primers. Our KASP primer set included two reverse primers: the reverse primer for *S. bicolor* was appended to the 5′ end with the complementary sequence of the FAM fluorophore quencher, while the reverse primer for *S. halepense* was appended to the 5′ end with the complementary sequence of the HEX fluorophore quencher. A universal forward primer was used in complement with these two primers (Table 1).

**Table 1:** Primers for single nucleotide polymorphism in the internal transcribed spacer (ITS) region for KASP PCR assays and PCR amplicon sequencing

Primer name	Primer sequence (5′‐3′)	Predicted melting temperature (° C)
ITS‐SNP591 (A/G)		
Universal FP	CGATTCGTGTCGGGCACAGC	67.4
*S. bicolor* RP	GGTCCTTAGGGCCGATGCA	65.9
*S. halepense* RP	GGTCCTTAGGGCCGATGCG	66.5
5’ FAM TAG (*S. bicolor*)	GAAGGTGACCAAGTTCATGCT	
5’ HEX TAG (*S. halepense*)	GAAGGTCGGAGTCAACGGATT	
ITS Primers		
*S. halepense* FP	GATTCTCAAGCTGGGCTGGTC	64.7
*S. halepense* RP	AATGGTCCGGTGAAGTGTTCG	64.6

### 2.4 KASP assay

A primer master mix, including the forward and both reverse primers, for ITS‐SNP591 was made according to the master mix manufacturer's recommendations (The Universal PACE Genotyping Master Mix 3CR Bioscience, Harlow, England, UK). This PCR master mix is compatible with most allele‐specific assays including both PACE and KASP. All primers were suspended in nuclease‐free water and a primer master mix was assembled with each of the species‐specific primers (18 μL each), the universal forward primer (45 μL), and nuclease‐free water (69 μL).

Reactions were assembled in 96‐well plates with the primer plus master mix combination (0.14 μL + 5 μL) and genomic DNA (4.86 μL at 10 ng μL^−1^). Reactions were performed in Bio‐Rad CFX96 Real‐Time System (Bio‐Rad Laboratories, Inc., Hercules, CA, USA). The protocol was performed as follows: activation at 94 °C for 15 min, followed by 10 touchdown cycles at 94 °C for 20 s, 64–56 °C for 1 min 10 s (dropping 0.8 °C per cycle), followed by 37 cycles at 94 °C for 20 s, 63 °C for 1 min 10 s, 37 °C for 1 min. An additional two cycles for insufficient clustering were performed at 94 °C for 20 s and 57 °C for 1 min when necessary. Florescence data was chosen for genotyping based on the cycle with the greatest difference between control populations without background amplification, this was determined to be cycles 26–28 of the amplification phase.

Positive control biotypes included a cultivated grain sorghum line (Rtx430) and a natural collection of *S. halepense*. Both biotypes consistently only amplified the FAM (*S. bicolor*) or HEX (*S. halepense*) fluorophore in the KASP assay, with negligible background fluorescence of the reciprocal fluorophore. KASP assays when typically employed produce clusters of samples corresponding to homozygotes of each allele and heterozygotes^24^; however, *Sorghum spp*. hybrids are not necessarily diploid, and the ITS region is multicopy in both species which leads to a spectrum of intermediate amplification that depends on the relative contribution of each parental phenotype to the hybrids genome. To account for this, ‘pseudohybrids’ controls were included which were generated to simulate hybrids between *S. bicolor* and *S. halepense* by mixing DNA from each control sample in varying ratios, ranging from 1:1 to 1:12, with increasing concentrations for both species. Florescence data from pseudohybrids were used to set the boundaries for what is called a true hybrid *versus* purely homozygous individuals.

### 2.5 Nanopore sequencing for validation

The KASP assay is designed to amplify a single SNP in a sequence; however, it is not normally used to distinguish a range of intermediate genotypes. To validate this assay's ability to correctly assign partial contributions of the two genotypes, we performed amplicon sequencing of the ITS with Nanopore sequencing. To design these primers, a 536 bp ITS sequence (GenBank: DQ005082.1) was analyzed for sequence homology against the *S. halepense* genome (Project ID# 1335880) using BLASTN (version 2.14.1). Coordinates corresponding to the top BLAST hit were extracted using Samtools (version 1.19.1^28^;) and 200 bp of flanking sequence on either side were included that resulted in a 936 bp fragment. This sequence was used to design PCR primers to amplify the 936 bp of the ITS region in the same biotypes that were run on the KASP assay above (Table 2).

**Table 2:** Comparison of KASP and Oxford Nanopore outputs for validation of KASP assay

Biotype*	KASP Output^†^		Nanopore Output^‡^		KASP	Nanopore	Genotype^§^
	FAM	HEX	T	C	θ	θ	
Arizona‐B	3.80	91.79	7	1599	0.0489	0.0044	*S. halepense*
Michigan‐D	4.39	85.05	5	1856	0.0351	0.0027	*S. halepense*
Ohio‐F	18.26	87.11	224	1956	0.2124	0.1140	Hybrid
Texas‐F	20.43	60.79	536	2251	0.3213	0.2338	Hybrid
Kansas‐H	28.62	55.60	813	1620	0.4816	0.4651	Hybrid
Texas‐A	35.34	39.69	1123	2054	0.7525	0.5003	Hybrid
Michigan‐H	45.76	39.52	503	437	0.8671	0.8555	Hybrid
Michigan‐A	62.60	28.47	1434	881	1.1295	1.0199	Hybrid
Maryland‐D	64.27	33.79	1726	1270	1.1010	0.9364	Hybrid
South Carolina‐C	82.73	13.85	1166	479	1.4108	1.1810	Hybrid
Rtx430	88.43	6.73	2507	39	1.4876	1.5552	*S. bicolor*
Pennsylvania‐B	96.14	6.17	1981	42	1.5117	1.5496	*S. bicolor*

* Subset from the MSU collection (Table S1).

^†^ Normalized fluorescence quantification from KASP assay.

^‡^ Raw nucleotide reads at SNP 591 in ITS region.

^§^ Genotype assigned by KASP assay.

The control biotypes, along with a subset from the MSU collection, were amplified using reactions (20 μL) consisting of 2X GoTaq G2 Green Master Mix (16 μL; Promega Corporation, Madison, WI, USA), each forward and reverse ITS primer (1 μL at 10 μM), and template DNA (2 μL at 20 ng μL^−1^). Cycling consisted of an initial denaturation step of 95 °C for 5 min, followed by 36 cycles of 95 °C for 30 s, 64 °C for 30 s, and 72 °C for 1.5 min, with a final extension of 72 °C for 5 min in a Bio‐Rad T100 thermal cycler (Bio‐Rad Laboratories, Inc., Hercules, CA, USA). Amplicons were cleaned with Thermo Scientific GeneJET PCR purification kit (Thermo Fisher Scientific, Waltham, MA, USA). Diluted products (≥ 20 ng μL^−1^) were sent for Nanopore sequencing (Oxford Nanopore Technologies, Oxford, England, UK). Raw base counts from Nanopore amplicon sequencing at the distinguishing SNP were evaluated and correlated with KASP fluorescence data.

### 2.6 Validation through greenhouse crosses and known/unknown biotypes

*S. bicolor*, *S. halepense*, and hybrid individuals were crossed under greenhouse conditions in the spring of 2024 at MSU to generate known hybrids. The known *S. bicolor* (Rtx430) and naturally collected *S. halepense* biotypes described above were used as parents, as well as a naturally collected hybrid. ‘Parent set one’ consisted of *S. halepense* (male) x *S. bicolor* (female), resulting seeds were collected from *S. bicolor*. ‘Parent set two’ consisted of *S. bicolor* (male) x *S. halepense* (female), resulting seeds were collected from *S. halepense*. ‘Parent set three’ consisted of *S. bicolor* (male) x hybrid (female), resulting seeds were collected from the hybrid. Seeds of each parent were sown in 25.5 × 25.5 cm flats, upon emergence of true leaves, seedlings were transplanted to 10.5 × 10.5 cm pots. Once seedlings reached adequate size, they were transplanted into 6.5 L pots for crossing. Upon seedhead formation and onset of pollen release, the respective biotypes were tied and seedheads bagged together. Excess individuals of each biotype were grown to ensure plants utilized in crosses were at the same developmental stage. Bagged seed heads were shaken daily to facilitate pollen flow between individuals. Seeds were harvested at maturity and grown for tissue collection and DNA extraction, following the methods outlined above. Due to the number of individuals collected from *S. bicolor* (female) parent in parent set one, DNA extractions were performed in pools of 20 individuals. All individuals from parent set two and three had DNA extracted from individuals.

Finally, we performed our assay on 62 *S. bicolor*, four *S. almum* biotypes [obtained from the NPGS *Sorghum* collection (Supporting Information, Table S2)], and 78 naturally collected *S. halepense* biotypes from the MSU collection (Supporting Information, Table S1). All biotypes were grown under greenhouse conditions and followed tissue collection and DNA extraction outlined above. Each individual was subjected to the KASP assay to assign their genotype.

### 2.7 Data analysis

**Figure 2:**
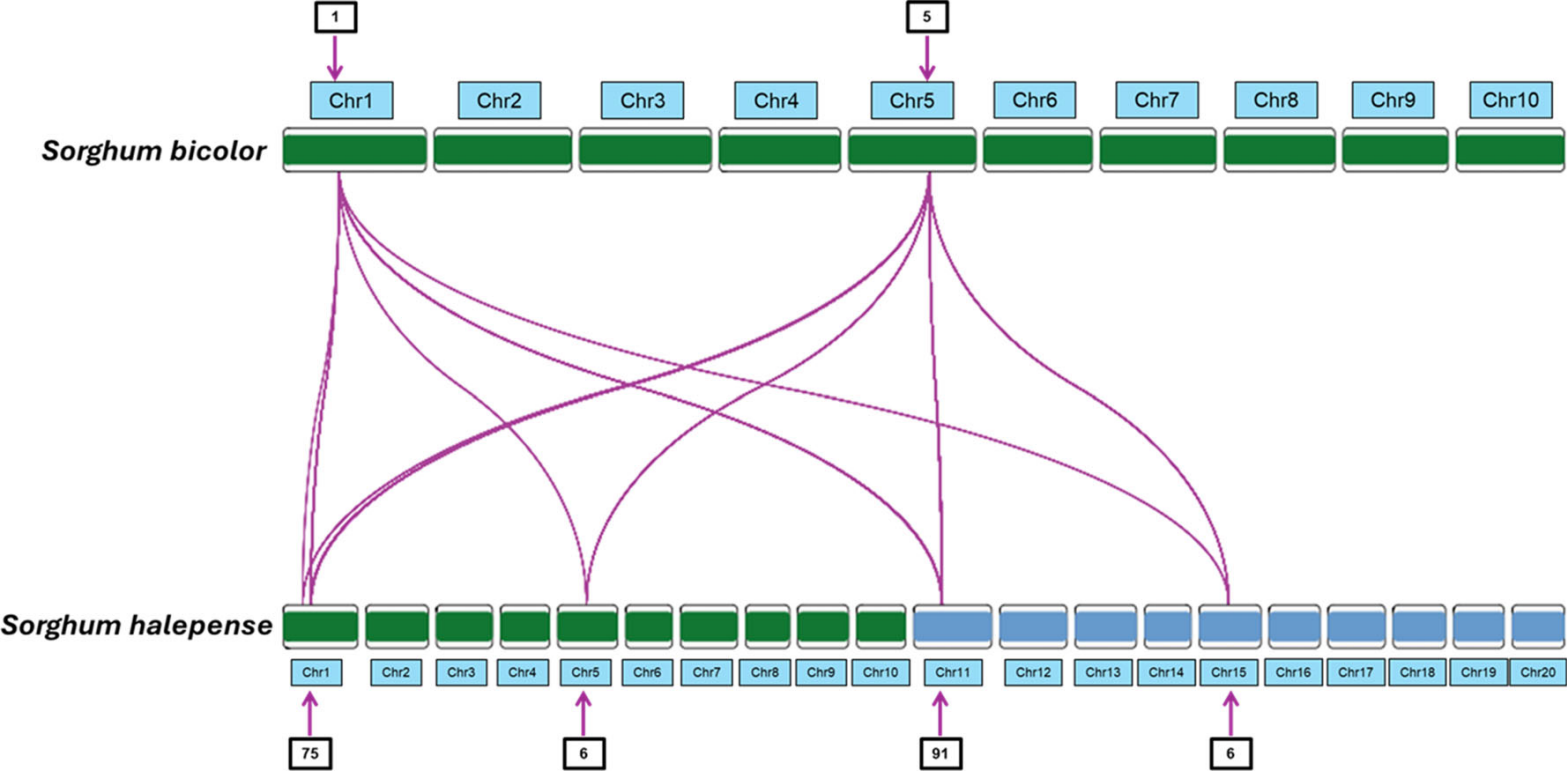
Ideogram of *Sorghum bicolor* and *S. halepense* alignment of the ITS region. Purple links represent shared synteny in the ITS region across chromosomes. Numbers in boxes above and below the ideogram indicate the number of ITS copies at each locus. Green highlighted chromosomes in *S. halepense* represent genome A, derived from *S. bicolor*.

A synteny analysis was performed using a 536 bp fragment of the ITS region for homology analysis using BLASTN (version 2.14.1) against the *S. bicolor* genome (GenBank: GCA_000003195.3). To determine the genomic location of the ITS sequences in *S. halepense*, sequences from the initial BLAST query against the *S. bicolor* genome, were used as BLAST queries against the *S. halepense* genome. Synteny plot was visualized using RIdeogram in R (version 4.3.3; Fig. 2).

**Figure 3:**
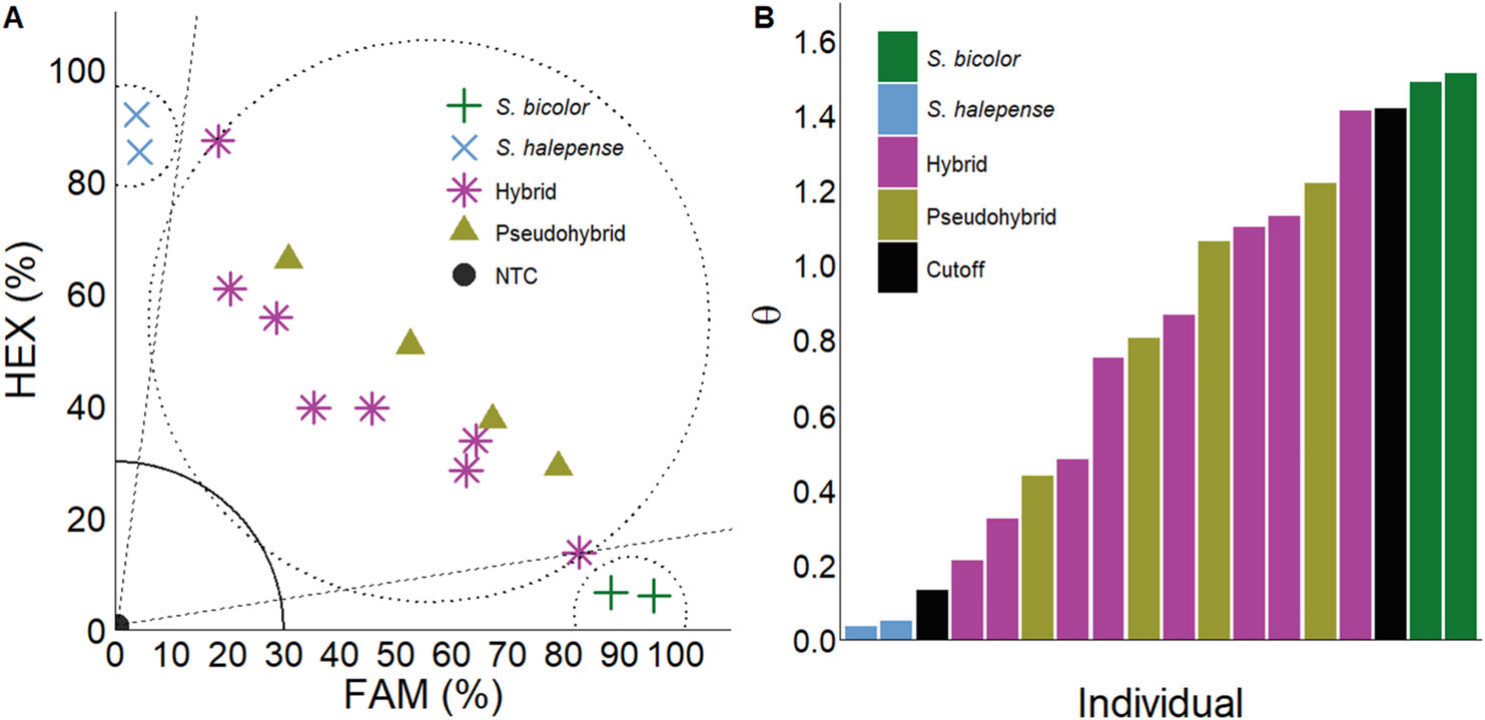
Reference KASP assay (A) of the wide range of genotypes between *S. bicolor* (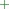), *S. halepense* (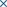), hybrid (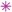), pseudohybrid (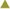), and no‐template (NTC●) controls. Dashed lines represent cutoffs for making genotyping assignments. Dashed circles enclose groupings based on genotype calls. The solid quarter circle line is the cutoff for no amplification. The arctan transformation (B) from the KASP assay plotted by individual assignment. Black bars in the arctan transformation note the cutoffs from the KASP assay.

Florescence data from the KASP assay was normalized by transforming the FAM and HEX florescence for each data point as a percentage of the maximum fluorescence for each fluorophore within a plate.^29^ Cutoffs for genotyping calls were made by calculating the geometric mean between the coordinate of outermost pseudohybrid controls used and either the control *S. bicolor* or *S. halepense*, then drawing a line from that point to the origin (0,0; Fig. 3). Additionally, a quarter circle at 30% was defined as the zone of no amplification.^24^ Sample genotypes were assigned based on the region of the plot where their fluorescence coordinates occurred. Pearson's correlation was conducted in R (version 4.3.2) on arctan transformed KASP (FAM/HEX) and Oxford Nanopore outputs (C/T) to cross‐validate KASP results.

## 3 RESULTS AND DISCUSSION

We have developed a simple, genetic assay that can accurately distinguish between *S. bicolor*, *halepense*, and their hybrids. Our KASP assay utilizes the SNP591 found in the ITS region that distinguished between these species and was consistent over spatial and genetic regions. The KASP assay has been validated through comparative sequencing methods, F_1_ individuals from greenhouse crosses (*n* = 1389), known (*n* = 66), and unknown biotypes (*n* = 78). Together, these approaches confirmed the effectiveness of our assay and evaluated it across diverse genetic and biological backgrounds.

### 3.1 Synteny analysis and KASP assay interpretation

*S. bicolor* contains ITS clusters with one copy on chromosome one and five copies on chromosome five. Whereas the tetraploid *S. halepense* contains 75 copies on chromosome one and six copies on chromosome five of subgenome A as well as 91 ITS copies on chromosome 11 and six copies on chromosome 15 of subgenome B. Synteny analysis revealed that the ITS in *S. bicolor* and *S. halepense* were homologous (Fig. 2). Duplication of ITS on chromosome 11 and 15 is expected for a tetraploid, *S. halepense*, formed from the original diploid *S. bicolor* x diploid *S. propinquum* hybridization event.^30^ The differences in duplication across chromosomes was surprising as chromosomes one and 11 possess over 93% of the copies compared to chromosomes five and 15. These observations aligned with the variable allele SNP frequencies we noted in our KASP assay (Fig. 3).

Additionally, our KASP primers' binding positions were analyzed in *S. bicolor* and *S. halepense* across all copies in both species. Our *S. halepense* primer in the KASP assay bound within each subgenome in *S. halepense*, is overwhelmingly bound within subgenome B on chromosome 11 with only one copy not binding and three copies on chromosome 15 binding with the *S. halepense* primer. Within subgenome A only one copy on chromosome one binds and three copies on chromosome five bind. Our *S. bicolor* primer in the KASP assay binds to one copy on chromosome one in *S. bicolor*.

Polyploid species, in this case hybrids between *S. bicolor* and *S. halepense* can display a range of intermediate amplifications of each fluorophore in a KASP assay due to there not being equal contributions of genetic information (i.e., target sequence), which produces a wide range of fluorescence coordinates within KASP assay plots (Fig. 3(A)). We artificially created pseudohybrids that mimic this wide range. Points that fall outside the bounds of the y‐ or x‐axis dashed lines were assigned as *S. halepense* or *S. bicolor*, respectively. Points that fall within the region bounded by the two dashed lines were assigned as a hybrid. The solid quarter circle line represents the zone of no amplification and is considered an unreliable region for genotyping; however, none of our samples ever fell in this region. To simplify KASP scatterplots in easy to interpret single values, arctan transformation graphs (Fig. 3(B)) have the individual sample along the x‐axis with the corresponding arctan value derived from the FAM/HEX ratio in the KASP assay. This transformation allows KASP results to be represented as single continuous variables for comparison.^29^ All KASP assays and arctan transformation graphs will be presented and interpreted using this approach.

### 3.2 Validation of the KASP assay through nanopore

**Figure 4:**
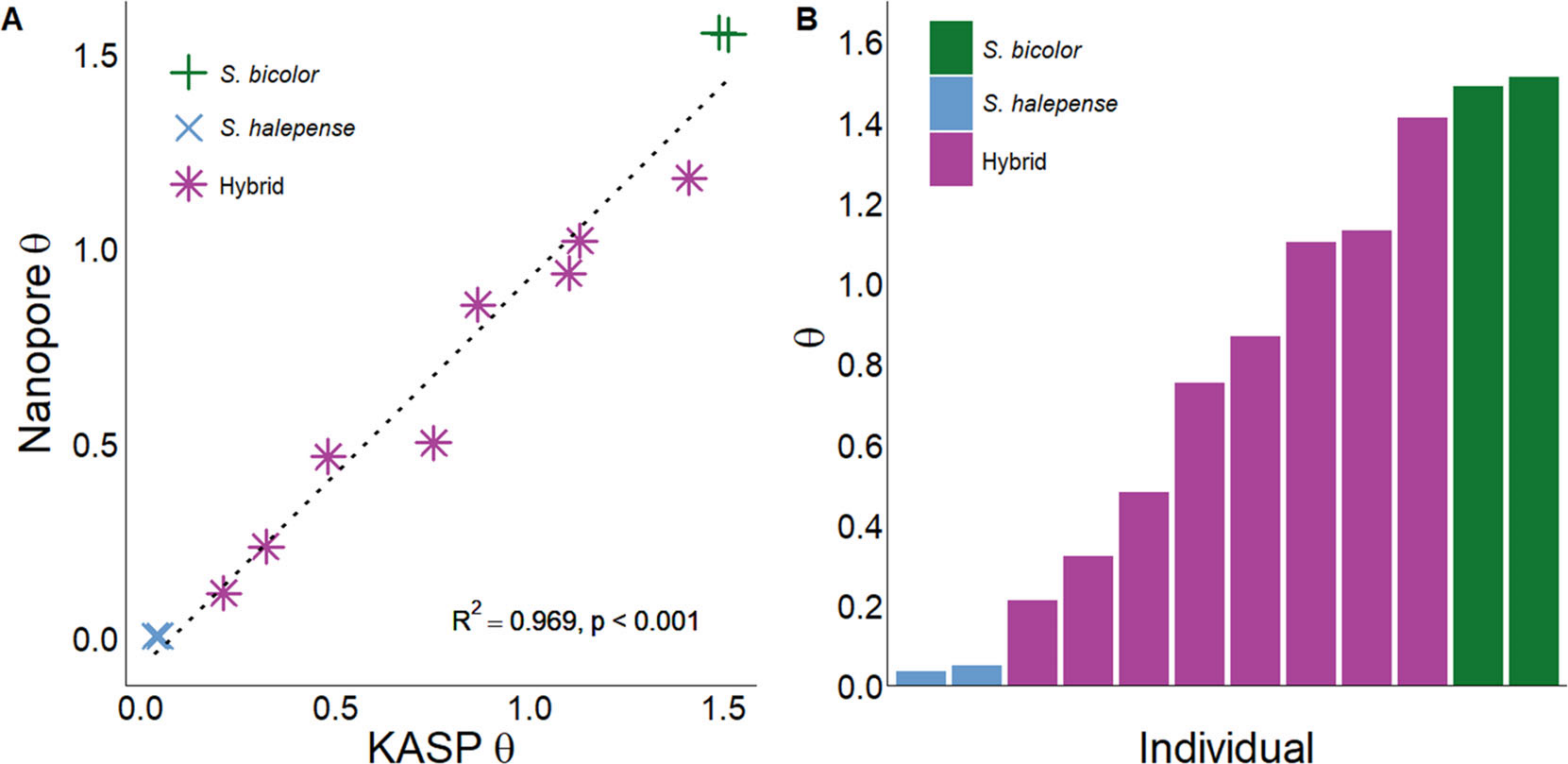
Correlation of arctan transformations (A) of the FAM/HEX ratio (KASP) and nucleotide ratio (Oxford Nanopore). *S. bicolor* (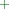), *S. halepense* (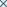), and hybrid (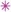). Significant positive relationship between the two methods (R^2^ = 0.97, *P* < 0.001). The arctan transformation (B) from the KASP assay of the same biotypes in (A).

Our data showed that the KASP assay performed as well as Nanopore sequencing for measuring SNP frequency and thus is a perfect proxy for genotyping *S. bicolor*, *S. halepense*, and their hybrids (R^2^ = 0.969, *P*‐value <0.001, Fig. 4). A similar finding was reported in sunflower (*Helianthus annuus* L.) when comparing KASP marker results and amplicon targeted sequencing,^31^ but did not show the specificity we established in our assay. Our study instead utilized Nanopore sequencing in order to quantify base calls at the distinguishing SNP site for comparison, which is indistinguishable from the fluorescence data from our KASP assay. Additionally, that study^31^ was working within a diploid, crop species with a high quality reference genome. The significance in our correlation in this assay is novel in a polyploid, weedy species.

### 3.3 Validation through F_1_ crosses

**Figure 5:**
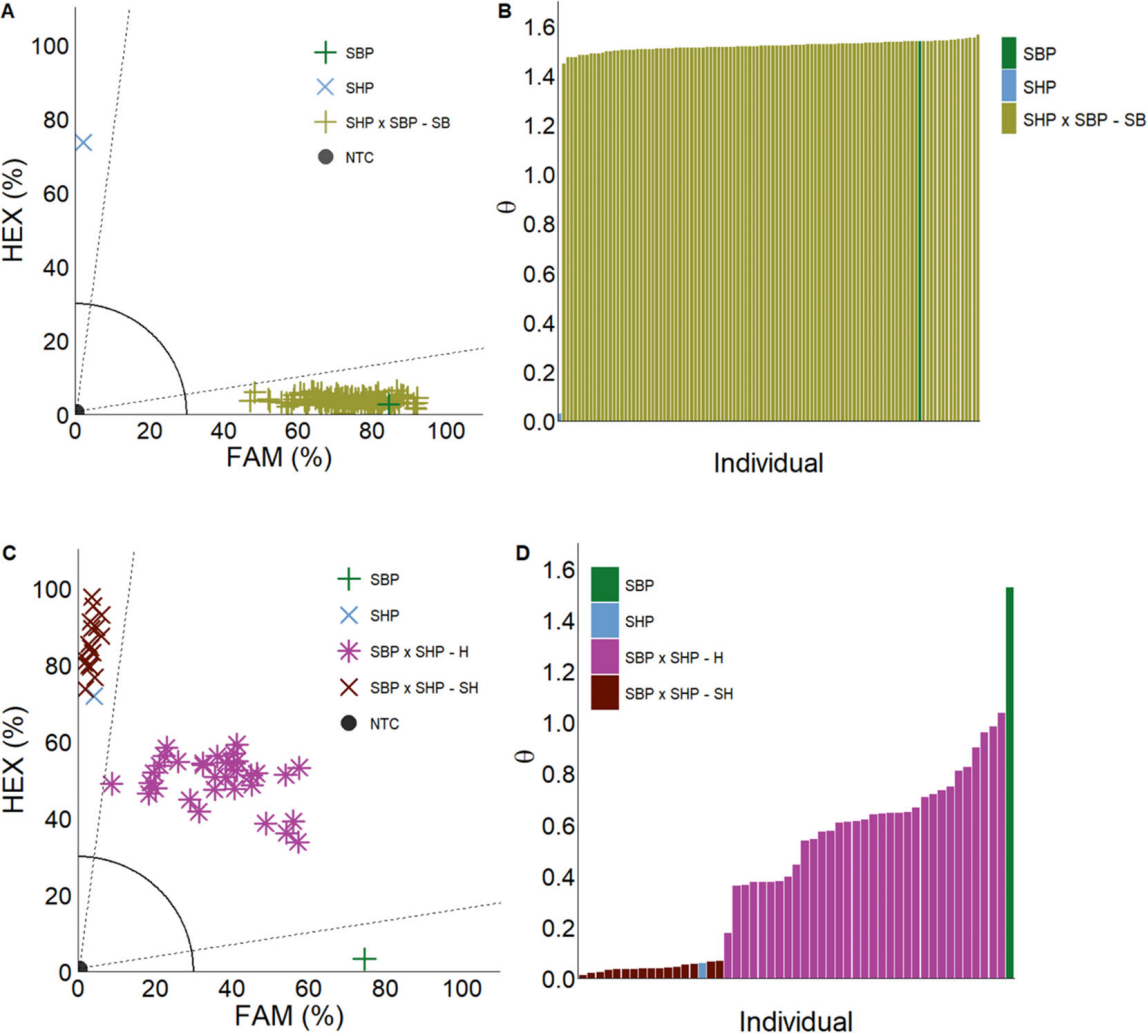
KASP assays and arctan transformation graphs from *S. bicolor* and *S. halepense* greenhouse crosses. Parent set 1 *S. bicolor* female parent (SBP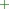), *S. halepense* male parent (SHP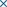), and F_1_ individuals assigned *S. bicolor* (SHP x SBP‐SB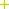) through KASP assay (A). Parent set 2 *S. halepense* female parent (SHP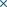), *S. bicolor* male parent (SBP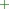), and F_1_ individuals assigned hybrid (SBP x SHP‐H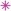) or *S. halepense* (SBP x SHP‐SH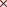) through KASP assay (C). Arctan transformation graph of each individual in the KASP assays, respectively, (B, D). NTC (●): no‐template control. Dashed lines represent cutoffs for making genotyping assignments. The solid quarter circle line is the cutoff for no amplification.

**Figure 6:**
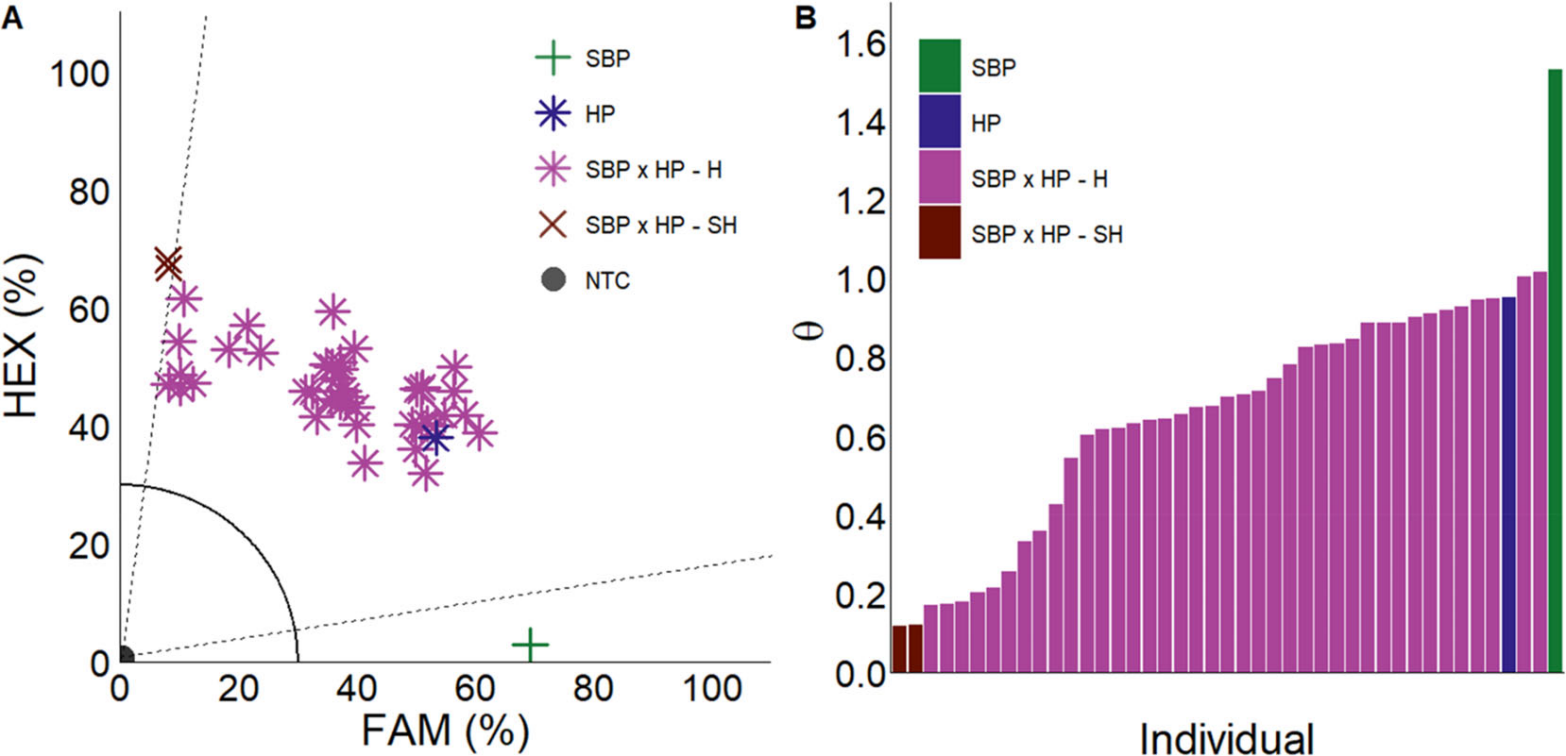
KASP assay and arctan transformation graphs from *S. bicolor* and hybrid greenhouse cross. Parent set 3 hybrid female parent (HP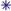), *S. bicolor* male parent (SBP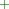), and F_1_ individuals assigned hybrid (SBP x HP‐H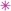) or *S. halepense* (SBP x HP‐SH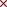) through KASP assay (A). Arctan transformation graph of each individual in KASP assay (B). NTC (●): no‐template control. Dashed lines represent cutoffs for making genotyping assignments. The solid quarter circle line is the cutoff for no amplification.

Greenhouse crosses revealed striking differences in hybrid formation, with *S. halepense* being more receptive to cross pollination than *S. bicolor*. No hybrids were formed when *S. bicolor* was the female (Fig. 5(A), (B)), while when *S. halepense* was the female, hybrids were formed (Fig. 5(C), (D)). Additionally, the hybrid parent was also more receptive to cross pollination than *S. bicolor* and displayed an allelic shift more towards HEX (*S. halepense*) than FAM (*S. bicolor*) (Fig. 6(A)).

Within parent set 1 (*S. halepense* male x *S. bicolor* female), all of the resulting F_1_ individuals were assigned *S. bicolor* (Fig. 5(A), (B)). Within parent set 2 (*S. bicolor* male x *S. halepense* female) 33% of the individuals were assigned as *S. halepense*, while the resulting 67% were assigned as hybrids (Fig. 5(C), (D)). Overall results in crosses between *S. bicolor* and *S. halepense* suggest that the *S. bicolor* parent utilized in this study is less receptive to cross pollination than its weedy relative. These crosses confirm crop‐to‐weed introgression observed in other studies.^30^, ^32^ Previous studies have shown a wide range of hybridization between *S. bicolor* and *S. halepense* depending on experimental design. Field studies have detected hybrid formation of 2 or 11% when *S. halepense* received pollen at 100or 5 meters from *S. bicolor*.^33^ Much lower hybrid formation (less than 2% across all genotypes) was found in a field study with uniform *S. halepense* infestation in a *S. bicolor* field (0.76 m row spacing) and *S. bicolor* genotypes as the pollen receivers.^10^ The *S. halepense* biotype used in our study did show significant hybrid formation when it was the pollen receiver (67%), which was much higher than the previous study reported^33^ most likely because the panicles were tied and bagged together from initial pollen release to mature seed.

Within parent set three (*S. bicolor* male x hybrid female), individuals from the female hybrid parent were assigned genotypes through our KASP assay (Fig. 6(A), (B)). Overall, 95% were genotyped as hybrid, 5% were assigned *S. halepense*, and no individuals were assigned as *S. bicolor*. Surprisingly, the hybrids that were formed from this cross demonstrated amplification closer to the HEX fluorophore, which corresponds more to *S. halepense*. One possible explanation involves the sheer overabundance of *S. halepense* ITS copies in comparison to *S. bicolor*. Previous greenhouse^34^ and field^10^ studies reported that the majority of hybrid seedlings were tetraploids. Given this factor, with the addition that the *S. halepense* KASP primer extensively amplifies subgenome B, hybrids are more biased towards *S. halepense*.

### 3.4 Validation through known and unknown collections

**Figure 7:**
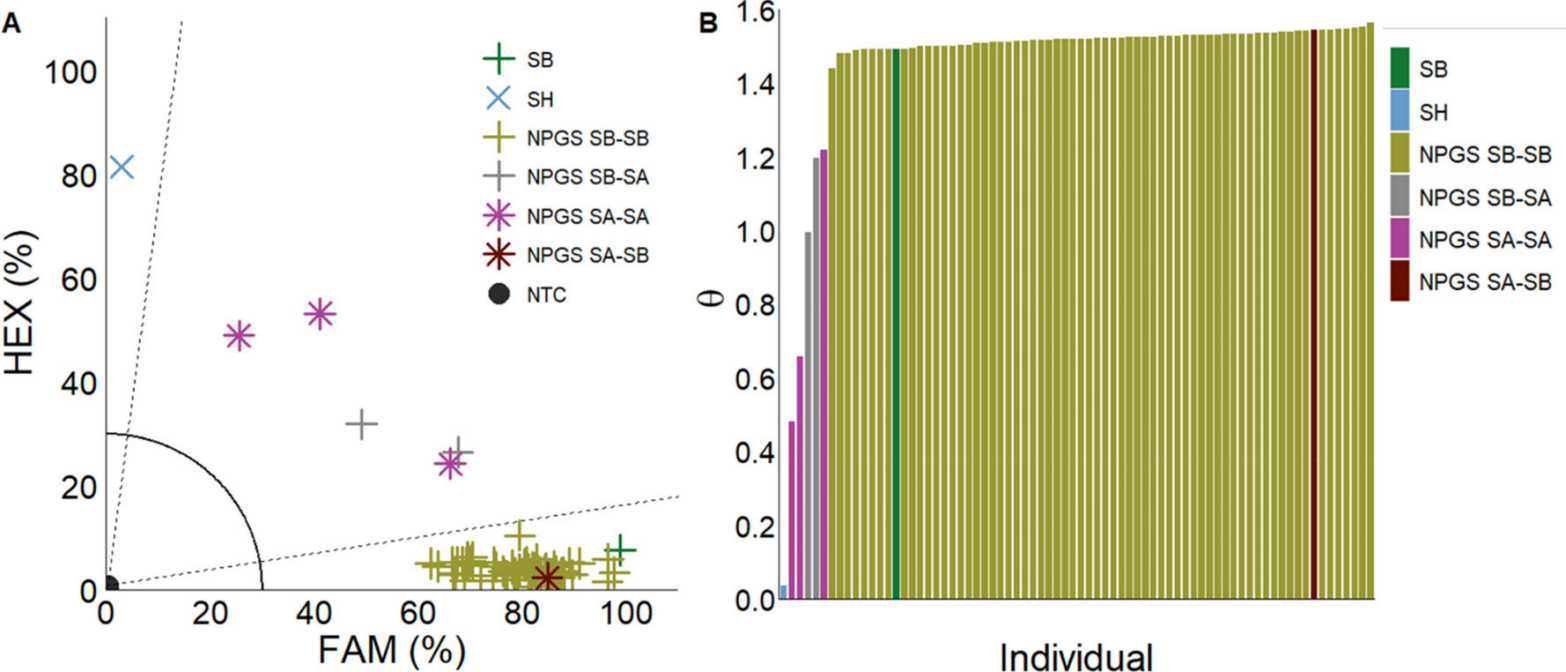
KASP assay (A) and arctan transformation graph (B) for NPGS *Sorghum* collection: *S. bicolor* control (SB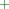), *S. halepense* control (SH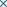), NPGS *S. almum* assigned *S. almum* (NPGS SA‐SA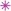), NPSG *S. almum* assigned *S. bicolor* (NPGS SA‐SB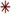), NPSG *S. bicolor* assigned *S. bicolor* (NPGS SB‐SB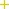), NPSG *S. bicolor* assigned *S. almum* (NPGS SB‐SA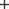), no‐template control (NTC●). Dashed lines represent cutoffs for making genotyping assignments. The solid quarter circle line is the cutoff for no amplification.

Sixty‐six biotypes from the NPGS *Sorghum* collection were assigned genotypes through our KASP assay (Fig. 7(A), (B)). Of the 62 that were *S. bicolor* lines, 97% aligned with the genotype given by NPGS, while 3% were assigned as hybrid through our KASP assay. The four NPGS *S. almum* lines, 75% aligned with the genotype given, while 25% were assigned as *S. bicolor* through our KASP assay. KASP results support the fact that hybrids between *S. bicolor* and *S. halepense* can be difficult to distinguish morphologically.^11^ A lack of genetic tools especially for outcrossing species may have led to misidentified individuals in germplasm collections. Studies differentiating species often utilize multiple SNPs within conserved regions like the ITS.^23^, ^24^ In contrast, our study successfully distinguished between *S. bicolor*, *S. halepense*, and their hybrids using a single SNP. This was validated by testing accessions that spanned genetic and spatial diversity, including biotypes from the United States to the *Sorghum* center of origin (present day Ethiopia).^35^ The consistency across these diverse accessions, supports this SNP marker to be of great significance between these two species.

**Figure 8:**
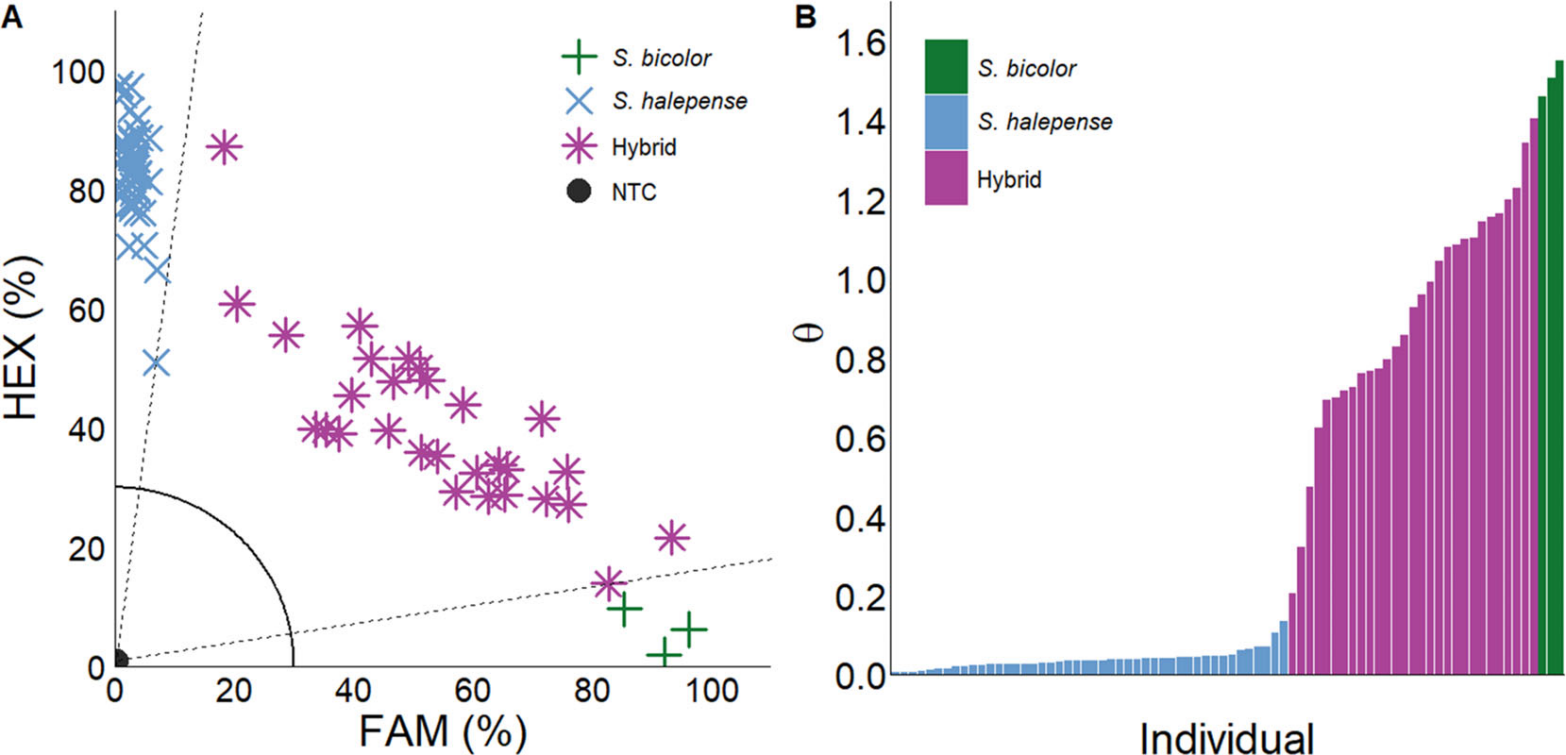
KASP assay (A) and arctan transformation graph (B) for unknown samples in MSU collection: *S. bicolor* (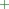), *S. halepense* (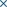), hybrid (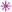), and no‐template controls (NTC●). Dashed lines represent cutoffs for making genotyping assignments. The solid quarter circle line is the cutoff for no amplification.

Seventy‐eight biotypes were collected across diverse regions of the United States and assigned genotypes through our KASP assay (Supporting Information, Table S2). Of the 78 biotypes collected, 59% were assigned *S. halepense*, 37% were assigned as a hybrid, and 4% were assigned *S. bicolor* (Fig. 8(A), (B)). Biotypes screened through the KASP assay were all collected under the assumption that they were *S. halepense* or had an intermediate phenotype between *S. bicolor* and *S. halepense*. These assignments show that a significant portion of the samples collected were indeed hybrids (37%), further supporting that hybrids are difficult to identify without genetic tools. This tool will also be valuable for examining the role of phenotypic plasticity in weeds. Often phenotypic plasticity is used to explain intraspecific variation; current genomic advances could help refine how we classify and interpret these differences within species. Many of these biotypes were sent and labelled as *S. halepense* seed stock from other universities and collaborators or collected under the same assumption.

The outcrossing rate under field conditions have been reported as less than 2% in male sterile *S. bicolor* parent lines pollinated by *S. halepense*.^10^ *Sorghum halepense* is expected to have a higher outcrossing rate than *S. bicolor* but still considered to be mostly self‐pollinated.^36^ These KASP assigned genotypes from diverse environments also suggest that there could be increased hybridization between these species than typically thought in the United States.

## 4 CONCLUSION

This study presents a novel, cost‐effective KASP assay capable of accurately distinguishing between *S. bicolor*, *S. halepense*, and their hybrids. By validating this assay through Nanopore sequencing, greenhouse crosses, and diverse germplasm, we demonstrated its utility across genetic backgrounds and environments. The detection of crop‐to‐weed introgression in greenhouse crosses and misidentified accessions notes the need for genetic tools. High frequency of hybridization in natural collections suggests more widespread hybridization than previously thought. This is the first study to provide a genetic tool that can accurately differentiate between *S. bicolor*, *S. halepense*, and their hybrids.

## Supporting information


**Data S1.** Supporting Information.

## Data Availability

The data that support the findings of this study are available from the corresponding author upon reasonable request.
